# The role of laboratory investigations in the classification of tremors

**DOI:** 10.1007/s10072-023-07108-w

**Published:** 2023-10-10

**Authors:** Luca Angelini, Roberta Terranova, Giulia Lazzeri, Kevin R E van den Berg, Michiel F Dirkx, Giulia Paparella

**Affiliations:** 1 Department of Human Neurosciences, Sapienza University of Rome, Viale Dell’Università 30, 00185 Rome, Italy; 2 Department of Medical, Surgical Sciences and Advanced Technologies “GF Ingrassia,” University of Catania, Catania, Italy; 3 IRCCS Ca’ Granda Ospedale Maggiore Policlinico, Neurology Unit, Milan, Italy; 4 Centre for Cognitive Neuroimaging, Donders Institute for Brain, Cognition and Behaviour, Radboud University, Nijmegen, The Netherlands; 5 Department of Neurology, Center of Expertise for Parkinson and Movement Disorders, Donders Institute for Brain, Cognition and Behaviour, Radboud University Medical Centre, Nijmegen, The Netherlands; 6 IRCCS Neuromed, Pozzilli (IS), Italy

**Keywords:** Tremor, Classification, Genetics, Neurophysiology, Neuroimaging

## Abstract

**Introduction:**

Tremor is the most common movement disorder. Although clinical examination plays a significant role in evaluating patients with tremor, laboratory tests are useful to classify tremors according to the recent two-axis approach proposed by the International Parkinson and Movement Disorders Society.

**Methods:**

In the present review, we will discuss the usefulness and applicability of the various diagnostic methods in classifying and diagnosing tremors. We will evaluate a number of techniques, including laboratory and genetic tests, neurophysiology, and neuroimaging. The role of newly introduced innovative tremor assessment methods will also be discussed.

**Results:**

Neurophysiology plays a crucial role in tremor definition and classification, and it can be useful for the identification of specific tremor syndromes. Laboratory and genetic tests and neuroimaging may be of paramount importance in identifying specific etiologies. Highly promising innovative technologies are being developed for both clinical and research purposes.

**Conclusions:**

Overall, laboratory investigations may support clinicians in the diagnostic process of tremor. Also, combining data from different techniques can help improve understanding of the pathophysiological bases underlying tremors and guide therapeutic management.

## Introduction

Tremor is defined as an involuntary, rhythmic, oscillatory movement of a body part [1] and is one of the most common movement disorders. It may occur as an isolated symptom, as is the case of essential tremor (ET), or it may be associated with other signs and symptoms, such as in Parkinson’s disease (PD). The current criteria for diagnosis and classification of tremors, as proposed by the International Parkinson and Movement Disorders Society (IPMDS), suggested the use of a two axes approach for tremor classification: axis 1, based on clinical features, whose evaluation would lead to the identification of a tremor syndrome, and axis 2, regarding tremor etiology [1].

Clinical assessment in several cases may not be sufficient for the diagnosis, and clinicians may be uncertain in defining whether they are observing tremor or another type of movement disorder. Moreover, in some cases the significance of additional signs besides tremor, including bradykinesia, dystonia, or ataxia, may also be uncertain. Laboratory tests, including measurements of serum and tissue biomarkers and neurophysiological and neuroimaging techniques, may therefore be helpful in the identification of tremors, better delineating a clinical syndrome and discovering the underlying etiology [2, 3]. However, the role of these tests in assisting clinicians in tremor classification is still debated. To the best of our knowledge, no work to date has comprehensively addressed the present issue.

This narrative review aims to examine the role of the various diagnostic laboratory methods in classifying tremors, following the two-axis approach suggested by the IPMDS Consensus Statement [1]. Individual techniques will be treated separately and organized into three main sections: serum and tissue biomarkers, electrophysiological and other neurophysiological investigations, and structural and receptor imaging [1]. We will also critically examine recent innovative methods for tremor assessment. Finally, we will discuss some future perspectives on the use of laboratory investigations.

## Serum and tissue biomarkers

The role of laboratory and genetic tests in axis 1 classification and tremor syndrome distinction is limited, whereas such tests may be of paramount importance in identifying specific etiologies in axis 2. The typical clinical features of some genetic syndromes manifesting with tremor are summarized in Table 1.

**Table 1:** Gene mutations and typical clinical manifestations of genetic syndromes manifesting with tremor

Disease	Gene mutations	Clinical manifestations
DYT-ANO3	ANO3 mutation (AD)	Isolated bibrachial action tremor, or various combinations of head, voice, and upper limbs tremor mainly manifesting during the disease course, associated with only minimal dystonic posturing
DYT-GCH1 (dopa-responsive dystonia)	GCH1 mutation (AD)	Upper-limb postural tremor, either alone or associated with parkinsonism, head, voice, chin, and parkinsonian rest tremor. Tremor characterized by dopamine response and diurnal fluctuations
DYT/PARK-TAF1 (Lubag disease)	TAF1 mutation (X-linked)	Head or limbs rest, postural and/or kinetic tremor, either prominent or combined with dystonia
SCA12	PPP2R2B mutation (AD)	Prominent or isolated upper-limb or head action tremor
SCA27	FGF14 mutation (AD)	Postural tremor as initial motor feature, associated with slowly progressive ataxia, orofacial dyskinesia, cognitive and psychiatric manifestations
FXTAS	FMR1 expansion	Bilateral upper-limb action tremor, more rarely unilateral, which may precede other clinical manifestations by years
PAPT	Mutation of several genes	Low-frequency (1—2 Hz) palatal tremor associated with progressive cerebellar dysfunction
Adult-onset paroxysmal head tremor responsive to acetazolamide	CACNA1A mutation and others	Discrete events of “no–no” head tremor lasting between 5 and 60 min and occurring several times per week, with progressive course
WD	ATP7B	“Wing-beating tremor” (high amplitude proximal tremor, elicited by sustained abduction of the arms, with flexed elbows and palms facing downward) is characteristic, but all kinds of movement disorders can be observed

*AD* autosomal dominant; *SCA12* spinocerebellar ataxia 12; *SCA27* spinocerebellar ataxia 27; *FXTAS* fragile X-associated tremor/ataxia syndrome; *PAPT* progressive ataxia and palatal tremor; *WD* Wilson’s disease

A first-line screening is important for all patients, whatever the specific tremor pattern, and should include: thyroid, liver and renal function tests, electrolytes (including calcium and magnesium), and blood cells count. Protein electrophoresis as well as screening for infectious diseases (such as HIV, syphilis, and borreliosis) can be based on the patient’s specific risk factors and clinical history. Laboratory tests can also support the diagnostic workup of specific genetic causes of tremors. These include serum ceruloplasmin in aceruloplasminemia and Wilson’s disease (WD), serum and urinary copper levels in WD, serum iron and ferritin in neuroferritinopathy and aceruloplasminemia, and sex hormone levels in sex chromosome aneuploidies.

Positive family history is frequently reported by ET patients, possibly suggesting an autosomal dominant inheritance pattern with incomplete penetrance. However, even though a long list of candidate loci and genes has been associated with ET [4], no genetic findings have conclusively been replicated. When tremor is classically part of a dystonic syndrome, dominant dystonias like DYT-ANO3 [5], DYT-GCH1 [6], X-linked dystonia-parkinsonism DYT/PARK-TAF1 (Lubag disease) [7] should be considered (Table 1). All dominant and recessive mutations causing genetic PD may be linked with combined tremor syndromes, with rest tremor being a cardinal feature. Rest tremor in familial PD is indistinguishable from the classic pill-rolling tremor of idiopathic PD [8]. On the other hand, different mutations associated with autosomal recessive PD, such as PRKN, PINK1, DJ1, etc., may manifest with primarily dystonic tremor (DT) [9]. As for diseases with main cerebellar involvement, some spinocerebellar ataxias (SCA), such as SCA12 [10] and SCA27 [11], can show tremor as a prominent symptom (Table 1). The combination of ataxia and tremor is also the clinical hallmark of fragile X-associated tremor/ataxia syndrome (FXTAS), a late-onset neurodegenerative disorder affecting predominantly males carrying a premutation allele in the FMR1 gene on chromosome X [12] (Table 1). Among other tremor syndromes, orthostatic tremor (OT) has been reported as a possible manifestation of very rare causes of spastic paraplegia (SPG), like SPG31 and SPG15 [13]. Rarely, some specific syndromic patterns, especially in the “tremor syndromes with prominent additional signs” category, can suggest a specific genetic or secondary etiology. This is the case of palatal tremor, that together with progressive ataxia can delineate the clinical picture of the so-called progressive ataxia with palatal tremor (PAPT), a rare clinical syndrome with several genetic disorders underlying familial cases, the most frequent of which being late-onset Alexander disease, SCA20, and POLG mutations [14]. Another specific clinical picture is the adult-onset paroxysmal head tremor responsive to acetazolamide [15] (Table 1).

Only a few genetic forms of tremor are treatable; the most relevant is WD, especially in young patients. It is caused by mutations in the ATP7B gene leading to impairment of a copper-transporting P-type ATPase, and tremor is one of the most common neurological manifestations, occurring in around 30–50% of patients [16] (Table 1).

Tremor can be a phenotypic manifestation of both neurodegeneration with brain iron accumulation (NBIAs) and primary familial brain calcifications (PFBC); however, the diagnostic suspicion of these disorders is much more frequently driven by specific imaging (see Table 3 below) rather than a particular tremor syndrome. Tremor is also a common manifestation of sex chromosome aneuploidies [17], including Klinefelter Syndrome (47, XXY karyotype), with up to 75% of affected patients showing bibrachial intention tremor, sometimes associated with head, voice, and leg tremor. Other rarer diseases like Jacobs syndrome (XYY), and supernumerary X or Y syndromes show generally more complex cognitive and psychological manifestations besides tremor.

## Electrophysiological and other neurophysiological investigations

Electromyography (EMG) and accelerometry (ACC) are the most used and available techniques for tremor assessment. EMG, recorded with surface or intramuscular needle electrodes, is used to detect the rhythmic entrainment of motor units [18], the hallmark of tremor, and is commonly applied in clinical and laboratory settings. Regarding ACC, inertial measurement units (IMU) are wearable sensors consisting of an accelerometer, a gyroscope, and often a magnetometer, used to capture the three-dimensional linear acceleration, angular velocity and space orientation of a body segment [19]. Some dedicated devices and smartphones apps have been developed to record body motion and tremor characteristics through motion transducers, although recording and analysis procedures are not standardized and many methodological factors may influence tremor evaluation [19].

Frequency analysis, such as the Fourier analysis, with the presence of a clear and narrow peak (≤ 2 Hz) is indicative of high rhythmicity and thus tremor [20]. A wider or unclear peak implies irregular motor unit firing, which should point toward other movement disorders, including myoclonus. Tremor frequency, expressed in cycles/seconds, can be estimated either directly by visually exploring the traces or indirectly through spectral analysis of EMG and ACC signals [18, 20–22]. The frequency of most pathological tremors is between 4 and 8 Hz, including ET and PD tremors [1, 23]. However, there is considerable overlap, which thus limits the use of tremor frequency evaluation for the differential diagnosis of tremor syndromes [22, 24]. Exceptions include OT (13–18 Hz), Holmes tremor (HT) (< 5 Hz) and myorhythmia (< 4 Hz) [1], where specific frequencies can be identified. In addition, analyzing the harmonics of the peak frequencies in the power spectrum, which characterize PD tremor, can help distinguish it from ET [25, 26]. Finally, both EMG and ACC can also be used to assess the regularity of tremor [20, 24, 27, 28] and identifying frequency variability, which is typical of DT, enhanced physiological tremor (EPT), and functional tremor (FT) [20, 23]. In this context, the so-called tremor stability index (TSI) has proven to be a useful tool to distinguish PD from ET tremor, as well as DT from ET; in particular, TSI in ET was found to be higher than PD but lower than DT [28, 29].

Tremor amplitude can be indirectly estimated by measuring the amplitude of the main frequency peak in the rectified and low-pass filtered power spectrum, i.e., demodulated EMG signal [30], or from ACC and gyroscopes data [18, 21]. It should be noted that ACC should be preferred to EMG to objectively compare tremor amplitude between patients, as it is less prone to technical artefacts (position of electrodes, muscle selection to be tested, etc.). Neurophysiological sensor-derived amplitude estimation is logarithmically related to tremor severity assessed by clinical rating scales [19, 31] and has been frequently used to assess treatment response objectively [18]. However, these methods all have a large inter-subject variability [3], and only a few applications exist in the differential diagnosis between the various tremor syndromes [24].

EMG and ACC can also record tremor changes in different activation conditions. For example, suppressing rest tremor with voluntary muscle activation (i.e., resetting) and a re-emergent component with a similar frequency a few seconds after posturing are typical features of PD tremor [20, 30, 32, 33]. The addition of weight loading on the upper limbs during laboratory tremor recordings influences the mechanical reflex component of tremor and can help distinguishing it from central neurogenic oscillation, leading to frequency reduction in EPT, while an amplitude increase is observed in FT [20, 23, 24, 34]; weight loading has also been used to investigate the presence of a central oscillating component in peripheral neuropathies which in some circumstances may be accompanied by tremor [35]. In this regard, standard nerve conduction studies help identify neuropathy when neuropathic tremor is suspected. A change in tremor frequency during contralateral arm rhythmic movements (i.e., entrainment) is typical of FT and included in the laboratory-supported criteria for the FT diagnosis [23, 36]; however, a new frequency peak at the tapping frequency in the power spectrum can suggest the presence of a mirror movement [20], which may be observed in DT and PD. Tremor suppression can be observed with contralateral tapping or ballistic movement in FT [36] (although this should be distinguished from the resetting phenomenon in PD tremor) or during the execution of sensory tricks in DT [20]. Mental tasks may increase PD tremor amplitude [20, 23].

A synchronous pattern of agonist and antagonist muscles has been observed in ET and can be used, with variable results, to distinguish rest tremor in ET from PD tremor, which is conversely characterized by an alternating pattern [22, 27]. A co-contraction of agonists and antagonists can be observed in dystonia and myoclonus [20, 24, 34]. High right-left intermuscular coherence and tonic coactivation are often seen in FT [36]. Moreover, EMG burst morphology and duration can be useful for the differential diagnosis with myoclonus (short burst < 50 ms in cortical and brainstem myoclonus) [37], and identification of Holmes tremor (longer bursts > 150 ms) and EPT (short bursts < 50 ms) [24]. Again, ACC signals may be used to measure tremor-associated signs in PD tremor or ET-plus [38]. Finally, the combination of other techniques with EMG and ACC, including electroencephalography and magnetoencephalography, allows to assess tremor coherence with cortical activity and evaluate the presence of cortical potentials by back-averaging, differentiating myoclonus and epileptic activity from tremor.

Several emerging neurophysiological techniques are not yet commonly used in clinical practice but have great potential for future application.

Optoelectronic systems detect the 3D displacement of different body parts and can provide objective information about tremor amplitude, frequency, body distribution and activation conditions [39–46], as well as tremor changes over time [45, 46] and possible alcohol and drug sensitivity of tremor [42, 47]. Video recordings, associated to computer analysis, are also useful in assessing movement features in a real-life context, and single-camera markerless motion capture systems have been recently developed. Machine learning on video data can help differentiate the different types of tremor, such as ET from PD, combining various tremor and movement parameters, including frequency and movement speed [48], and ET from FT through the analysis of tremor entrainment [49]. Finally, both optoelectronic and video kinematic analyses can be used for the evaluation of neurologic signs associated with tremor, including bradykinesia [41, 43–45, 50, 51] and possible gait disturbances [52], helping in defying tremor in the context of ET-plus, PD and other conditions.

Digitizing tablets have been widely used to analyze tremors during handwriting and drawing tasks (e.g., Archimedes’ spiral) [18, 53]. Data can also be derived from sensors fixed on the subject’s hand or the pen, and combinations of drawing analysis with motion transducers and magnetic resonance imaging (MRI) are promising approaches for tremor assessment. Beside tremor frequency and amplitude, these analyses provide information on movement regularity and line orientation and can help distinguish DT patients, which show less variability and less clearly identifiable tremor orientation axis in spiral drawing, from FT and ET [54, 55]. Another possible application of drawing analysis is the longitudinal assessment of tremor [56], and the detection of additional signs to tremor, i.e. micrographia and movement slowness [57, 58], as well as drawing ataxia [59].

Voice analysis can quantify tremor by analyzing rhythmic fluctuation of the fundamental frequency and sound pressure level, and both spectral analysis for frequency detection or machine learning methods can be applied [18, 60]. Frequency variability and diadochokinesis is useful in distinguishing tremor from spasmodic dysphonia (SD) and amyotrophic lateral sclerosis (ALS); in particular, patients with voice tremor showed a higher variability of the fundamental frequency than ALS patients, and greater diadochokinesis (i.e., slower and more irregular syllable repetition) was observed in patients with tremor and ALS then in those with SD [61]. The distinction between voice tremor and SD can also make use of needle EMG for the identification of larynx rhythmic muscle contraction at a 4–7 Hz frequency typical of ET [62] and laryngoscopy for the visualization of tremor in the palate, pharynx and larynx during specific tasks [63].

Other neurophysiological techniques mainly used in laboratory settings include the study of the blink reflex recovery cycle, which measures brainstem excitability often altered in PD, DT, and ET-plus with rest tremor but not in ET patients [64–66]. The somatosensory temporal discrimination threshold is altered in patients with dystonia, and its measurement can help distinguish ET, isolated head tremor and DT [67–69]. Presynaptic inhibition between antagonist muscles of the forearm, measured through the evaluation of electrically conditioned H reflex, is altered in cervical dystonia with tremor but normal in ET [70]. Finally, tremor reset induced by transcranial magnetic stimulation applied to the motor cortex or cerebellum may provide clues to the distinction of ET from PD tremor and DT [71–73].

The main neurophysiological techniques and findings useful in classifying the major tremor syndromes are summarized in Table 2.

**Table 2:** Main neurophysiological techniques and findings useful in the classification of the major tremor syndromes

Syndrome	Neurophysiological techniques and major findings
ET	EMG/ACC: synchronous pattern between agonist and antagonist muscles activity, high coherence between ipsilateral but not side-to-side muscles, high TSI (> 1.05) Drawing analysis: identifiable tremor orientation axis in spiral drawing
ET-plus	ACC, optoelectronic and video recordings: objective assessment of soft signs, e.g., bradykinesia and gait disturbances Drawing analysis: bradykinesia, micrographia and drawing ataxia BRrc: increased R2 component in ET-plus with rest tremor
EPT	EMG/ACC: frequency variability, short EMG bursts (< 50 ms), frequency reduction with weight loading
OT	EMG/ACC: typical frequency (13–18 Hz), high right-left coherence
DT	EMG/ACC: frequency variability, co-contraction of agonist and antagonist muscles, appearance of a new frequency peak during contralateral arm rhythmic movements when mirror movements are present, tremor suppression during sensory tricks BRrc: increased R2 component STDT: altered Presynaptic inhibition between antagonist forearm muscles: altered
PT	EMG/ACC: suppression of rest tremor with voluntary muscle activation, reemergent tremor, amplitude increase with mental tasks, presence of harmonics of the main peak in the power spectrum, low TSI (< 1.05) BRrc: increased R2 component
FT	EMG/ACC: frequency variability, high right-left coherence and tonic coactivation at tremor onset, amplitude increase with weight loading, change in tremor frequency, or tremor suppression during contralateral arm rhythmic movements, tremor suppression with contralateral arm ballistic movements Drawing analysis: variability in spiral drawing
VT	EMG: frequency 4–7 Hz Laryngoscopy: visualization of tremor in the palate, pharynx, and larynx

*ET* essential tremor; *ET-plus* essential tremor plus; *EPT* enhanced physiological tremor; *OT* orthostatic tremor; *DT* dystonic tremor; *PT* parkinsonism associated tremor; *FT* functional tremor; *VT* voice tremor; *BRrc* blink reflex recovery cycle; *EMG* electromyography; *ACC* accelerometry; *STDT* somatosensory temporal discrimination threshold

## Structural and receptor imaging

Overall, techniques based on receptor imaging are mainly used to distinguish tremor syndromes (axis 1), while structural imaging is mainly used to establish tremor etiology (axis 2).

The most widely used receptor imaging technique is the 123I-FP-CIT single photon emission computerized technology, i.e., dopamine transporter SPECT (DaT-SPECT), which detects nigrostriatal degeneration. This has proven useful in axis 1 classification when considering tremor in PD vs ET and DT [74]. The DaT-SPECT scan is typically considered negative in ET, although the present data is not entirely conclusive [75]. DaT-SPECT can also contribute to the axis 2 classification as it may distinguish parkinsonian tremors, which usually have an abnormal DaT-SPECT, from parkinsonian tremor due to drug-induced parkinsonism, that usually shows normal DaT-SPECT results [76].

Structural imaging can be used to determine a specific etiology (axis 2) and is generally indicated in case of combined tremor syndromes where tremor is 1) focal/unilateral, 2) non-classical in appearance, 3) in case there is a sudden onset or stepwise deterioration or 4) a family history of movement disorders combined with cognitive or psychiatric symptoms [34]. MRI is the preferred method, although computerized tomography (CT) may play a role in assessing tremors in emergency settings. Furthermore, CT can provide information in case of cerebral calcifications, which may indicate numerous metabolic or genetic diseases causing tremor in combination with other neurological signs. Typical MRI patterns indicating specific etiologies include cerebellar lesions or atrophy in intention tremor syndrome due to cerebellar dysfunction, signs pointing towards parkinsonian syndromes (e.g., the hummingbird sign in progressive supranuclear palsy (PSP)), basal ganglia hyperintensities on T2 MRI in WD [77], lesions of the red nucleus, thalamus, nigrostriatal tract, pons and superior cerebellar peduncle in Huntington’s disease (HT) [78]. Other tremor conditions in which neuroimaging can make a key contribution are summarized in Table 3 [77, 79–84].

**Table 3:** Neuroradiological findings typical of various diseases with specific etiologies that may present initially or predominantly with tremor

Disease	Neuroimaging findings
Neuroferrinopathy	Iron deposition observed as low-intensity areas on T2WI and as signal loss on T2 ∗ WI MRI in widespread location including globus pallidus, putamen, and dentate nuclei accompanied by lesions in the caudate nuclei or thalami, and cortical deposition; tissue edema and gliosis hyperintense areas on T2WI MRI in the same regions of iron deposition; symmetrical cystic changes in the basal ganglia in the advanced stages; atrophy in cerebellar and cerebral cortices (frontal lobe)
PKAN	Iron deposition observed as hypointensity with an area of central hyperintensity in the globus pallidi on T2WI and T2*WI MRI (“eye-of-the-tiger” sign); abnormalities restricted to the globus pallidus and substantia nigra
Aceruloplasminemia	Iron deposition observed as hypointensity on T2WI and T2*WI MRI with distribution comparable to neuroferritinopathy, but all basal ganglia nuclei and thalami simultaneously and homogenously involved
SCA12	Cortical cerebellar atrophy, eventually associated with cerebral atrophy, white matter changes and PET hypometabolism in cerebral cortex
SCA27	Normal or cortical cerebellar atrophy and PET cerebral and cerebellar hypometabolism
PFBC	Bilateral calcification of the basal ganglia with possible involvement of other brain regions, observed as hyperdense lesions in CT and low intensity signal on a T2WI and low or high intensity signals on T1WI MRI
WD	Cortical, brainstem and cerebellar atrophy, MRI diffuse white matter hyperintensities (WMHs), T2WI hyperintensity of the putamina, increased susceptibility and diffusion-related abnormalities
FXTAS	Symmetric T2WI hyperintensity of middle cerebellar peduncles and peridentate white matter, cerebral white matter, and corpus callosum; cerebellar cortex, cerebral and corpus callosum atrophy; SPECT decreased uptake of the dopamine transporter and post-synaptic D2 receptors in the striata
PAPT	Sporadic: inferior olives hypertrophy and T2WI hyperintensity. Familial: brainstem and cervical cord atrophy

*MRI* magnetic resonance imaging; *CT* computerized tomography; *PET* positron emission tomography; *SPECT* single-photon emission computed tomography; *T1WI* T1 weighted image; *T2WI* T2 weighted image; T2*WI T2* weighted image; *WMHs* white matter hyperintensities; *D2* dopamine receptors D2; *PKAN* pantothenate kinase-associated neurodegeneration; *SCA12* spinocerebellar ataxia 12; *SCA27* spinocerebellar ataxia 27; *PFBC* primary familial brain calcifications; *WD* Wilson’s disease; *FXTAS* fragile X-associated tremor/ataxia syndrome; *PAPT* progressive ataxia and palatal tremor

There are several other imaging methods reported in the literature that, to date, are not commonly used in clinical practice. Data from MRI relaxometry [85], machine learning analysis of structural measures [86], DTI measures of basal ganglia and cerebellum [87], neuromelanin and nigrosome-1 imaging [88, 89], substantia nigra hyperechogenicity in transcranial sonography [90], and cardiac 123metaiodobenzylguanidine (MIGB) scintigraphy [91] have been mainly applied to distinguish ET and PD. Structural, functional, and perfusion imaging have been used for research purposes for the differential diagnosis between ET and DT [92, 93]. Though not directly compared within one study, distinct tremor-related cerebellar activation patterns have been identified between ET, DT and PD [94]. Namely, in ET reduced cerebellar task-related activity, resting state connectivity, and cerebellar atrophy have been demonstrated [95]. In DT, where the cerebellar vermis/fastigial nuclei loop is preferentially involved, the cerebellum’s role must be included within a larger network involving the basal ganglia and cortical motor regions. In PD, the cerebellum is likely involved in specific tremor subtypes, i.e., postural tremor and dopamine-resistant rest tremor [95, 96]. ET and ET-plus may also potentially be distinguished, considering white matter abnormalities [97] and functional connectivity in areas outside the cerebello-thalamo-cortical circuit [98]. ET and OT differ in terms of cortical and subcortical grey matter volume [99]. Again, focal voice tremor and dystonic voice tremor show different brain volume data [100]. Finally, neuroimaging may provide information on the disease course, showing signs of neurodegeneration such as cortical atrophy [101], and on the response of tremor to therapy [96].

## Conclusions and future perspectives

Neurophysiology plays an important role in tremor definition and axis 1 classification; it is useful in objectively measuring tremor parameters, tremor evolution over time, detecting elements not visible to the naked eye, and providing crucial elements in the definition of specific syndromes, especially when clinical manifestations overlap. On the other hand, serum and genetic tests and neuroimaging, although providing some clues for the differential diagnosis of the tremor syndromes, play their main role in identifying axis 2 etiologies. These methods must be preceded by a thorough clinical evaluation, which aims to select the most useful ancillary tests and guide the interpretation of the results.

Future studies on techniques for tremor assessment should directly compare different groups of patients, focusing on identifying helpful elements to distinguish syndromes and etiologies. This is particularly relevant for syndromes with similar manifestations, where a correct diagnosis is necessary to optimize clinical management and for a more accurate classification for research needs. Again, although many of the technologies described are not yet integrated into clinical practice, developing innovative technologies is highly promising for the near future. Thanks to wearable devices and recent developments in telemedicine, for instance, continuous, non-invasive, accurate, domestic monitoring of symptoms will be possible, with relevant implications for patient management. Moreover, developments in neuroimaging and neurophysiology are contributing to an increasingly deeper pathophysiological understanding of tremor in different conditions, and this may lead in the future to further classifications based on underlying mechanisms in the perspective of increasingly accurate targeted therapies.

## Data Availability

Data sharing is not applicable to this article as no datasets were generated or analysed during the current study.

## Abbreviations

*ACC*: Accelerometry
*AD*: Autosomal dominant
*ALS*: Amyotrophic lateral sclerosis
*CT*: Computerized tomography
*D2*: Dopamine receptors D2
*DaT-SPECT*: Dopamine transporter single-photon emission computed tomography
*DT*: Dystonic tremor
*DYT/PARK-TAF1*: Dystonia-parkinsonism due to mutations in TAF1 gene
*DYT-ANO3*: Dystonia due to mutations in ANO3 gene
*DYT-GCH1*: Dystonia due to mutations in GCH1 gene
*EMG*: Electromyography
*EPT*: Enhanced physiological tremor
*ET*: Essential tremor
*FT*: Functional tremor
*FXTAS*: Fragile X-associated tremor/ataxia syndrome
*HT*: Holmes tremor
*IMU*: Inertial measurement units
*IPMDS*: International Parkinson and Movement Disorders Society
*MIBG*: Metaiodobenzylguanidine
*MRI*: Magnetic resonance imaging
*NBIA*: Neurodegeneration with brain iron accumulation
*OT*: Orthostatic tremor
*PAPT*: Progressive ataxia and palatal tremor
*PD*: Parkinson’s disease
*PET*: Positron emission tomography
*PFBC*: Primary familial brain calcifications
*PKAN*: Pantothenate kinase-associated neurodegeneration
*PSP*: Progressive supranuclear palsy
*SCA12*: Spinocerebellar ataxia 12
*SCA20*: Spinocerebellar ataxia 20
*SCA27*: Spinocerebellar ataxia 27
*SD*: Spasmodic dysphonia
*SPG15*: Spastic paraplegia 15
*SPG31*: Spastic paraplegia 31
*T1WI*: T1 weighted image
*T2*WI*: T2* weighted image
*T2WI*: T2 weighted image
*TSI*: Tremor stability index
*WD*: Wilson’s disease
*WMHs*: White matter hyperintensities

## References

[1] Bhatia KP, Bain P, Bajaj N (2018). Consensus Statement on the Classification of Tremors. From the Task Force on Tremor of the International Parkinson and Movement Disorder Society. Mov Disord.

[2] Marsili L, Bologna M, Mahajan A (2023). Diagnostic Uncertainties in Tremor. Semin Neurol.

[3] Elble RJ, Ondo W (2022). Tremor rating scales and laboratory tools for assessing tremor. J Neurol Sci.

[4] Magrinelli F, Latorre A, Balint B (2020). Isolated and combined genetic tremor syndromes: a critical appraisal based on the 2018 MDS criteria. Parkinsonism Relat Disord.

[5] Stamelou M, Charlesworth G, Cordivari C (2014). The phenotypic spectrum of DYT24 due to ANO3 mutations. Mov Disord.

[6] Trender-Gerhard I, Sweeney MG, Schwingenschuh P (2009). Autosomal-dominant GTPCH1-deficient DRD: clinical characteristics and long-term outcome of 34 patients. J Neurol Neurosurg Psychiatry.

[7] Evidente VGH, Advincula J, Esteban R (2002). Phenomenology of “Lubag” or X-linked dystonia-parkinsonism. Mov Disord.

[8] Greenland JC, Barker RA (2018) The Differential diagnosis of parkinson’s disease. In: Stoker TB, Greenland JC, (eds) Parkinson’s disease: pathogenesis and clinical aspects [Internet]. Codon Publications, Brisbane, AU

[9] Schneider SA, Bhatia KP, Hardy J (2009). Complicated recessive dystonia parkinsonism syndromes. Mov Disord.

[10] Choudhury S, Chatterjee S, Chatterjee K (2018). Clinical Characterization of Genetically Diagnosed Cases of Spinocerebellar Ataxia Type 12 from India. Mov Disord Clin Pract.

[11] Groth CL, Berman BD (2018). Spinocerebellar Ataxia 27: A Review and Characterization of an Evolving Phenotype. Tremor Other Hyperkinet Mov (N Y).

[12] Berry-Kravis E, Abrams L, Coffey SM (2007). Fragile X-associated tremor/ataxia syndrome: clinical features, genetics, and testing guidelines. Mov Disord.

[13] Picillo M, Erro R, Munhoz RP, Fasano A (2018). When shaking during standing points to hereditary spastic paraplegias. Parkinsonism Relat Disord.

[14] Erro R, Reich SG (2022). Rare tremors and tremors occurring in other neurological disorders. J Neurol Sci.

[15] Geerlings RPJ, Koehler PJ, Haane DYP (2011). Head tremor related to CACNA1A mutations. Cephalalgia.

[16] Maciel R, Portela DC, Xavier-Souza G (2019). Improvement of “Wing-Beating” Tremor in Wilson’s Disease With High Dose of Zolpidem: A Case Report. Mov Disord Clin Pract.

[17] Carvalho V, Ferreira JJ, Correia Guedes L (2021). Tremor and Parkinsonism in Chromosomopathies - A Systematic Review. Mov Disord.

[18] Haubenberger D, Abbruzzese G, Bain PG (2016). Transducer-based evaluation of tremor. Mov Disord.

[19] Elble RJ, McNames J (2016). Using Portable Transducers to Measure Tremor Severity. Tremor Other Hyperkinet Mov (N Y).

[20] Deuschl G, Becktepe JS, Dirkx M (2022). The clinical and electrophysiological investigation of tremor. Clin Neurophysiol.

[21] Vescio B, Quattrone A, Nisticò R (2021). Wearable Devices for Assessment of Tremor. Front Neurol.

[22] Nisticò R, Pirritano D, Salsone M (2011). Synchronous pattern distinguishes resting tremor associated with essential tremor from rest tremor of Parkinson’s disease. Parkinsonism Relat Disord.

[23] van der Stouwe AMM, Elting JW, van der Hoeven JH (2016). How typical are “typical” tremor characteristics? Sensitivity and specificity of five tremor phenomena. Parkinsonism Relat Disord.

[24] van der Veen S, Klamer MR, Elting JWJ (2021). The diagnostic value of clinical neurophysiology in hyperkinetic movement disorders: A systematic review. Parkinsonism Relat Disord.

[25] Muthuraman M, Hossen A, Heute U (2011). A new diagnostic test to distinguish tremulous Parkinson’s disease from advanced essential tremor. Mov Disord.

[26] Wile DJ, Ranawaya R, Kiss ZHT (2014). Smart watch accelerometry for analysis and diagnosis of tremor. J Neurosci Methods.

[27] Nisticò R, Quattrone A, Crasà M (2022). Evaluation of rest tremor in different positions in Parkinson’s disease and essential tremor plus. Neurol Sci.

[28] di Biase L, Brittain J-S, Shah SA (2017). Tremor stability index: a new tool for differential diagnosis in tremor syndromes. Brain.

[29] Panyakaew P, Cho HJ, Lee SW (2020). The Pathophysiology of Dystonic Tremors and Comparison With Essential Tremor. J Neurosci.

[30] Vial F, Kassavetis P, Merchant S (2019). How to do an electrophysiological study of tremor. Clin Neurophysiol Pract.

[31] Giuffrida JP, Riley DE, Maddux BN, Heldman DA (2009). Clinically deployable Kinesia technology for automated tremor assessment. Mov Disord.

[32] Dirkx MF, Zach H, Bloem BR (2018). The nature of postural tremor in Parkinson disease. Neurology.

[33] Leodori G, Belvisi D, De Bartolo MI (2020). Re-emergent Tremor in Parkinson’s Disease: The Role of the Motor Cortex. Mov Disord.

[34] van de Wardt J, van der Stouwe AMM, Dirkx M (2020). Systematic clinical approach for diagnosing upper limb tremor. J Neurol Neurosurg Psychiatry.

[35] Silsby M, Fois AF, Yiannikas C (2023). Chronic inflammatory demyelinating polyradiculoneuropathy-associated tremor: Phenotype and pathogenesis. Eur J Neurol.

[36] Schwingenschuh P, Saifee TA, Katschnig-Winter P (2016). Validation of “laboratory-supported” criteria for functional (psychogenic) tremor. Mov Disord.

[37] Merchant SHI, Vial-Undurraga F, Leodori G (2020). Myoclonus: An Electrophysiological Diagnosis. Mov Disord Clin Pract.

[38] Monje MHG, Foffani G, Obeso J, Sánchez-Ferro Á (2019). New Sensor and Wearable Technologies to Aid in the Diagnosis and Treatment Monitoring of Parkinson’s Disease. Annu Rev Biomed Eng.

[39] Bologna M, Berardelli I, Paparella G (2019). Tremor Distribution and the Variable Clinical Presentation of Essential Tremor. Cerebellum.

[40] Deuschl G, Wenzelburger R, Löffler K (2000). Essential tremor and cerebellar dysfunction Clinical and kinematic analysis of intention tremor. Brain.

[41] Bologna M, Paparella G, Colella D (2020). Is there evidence of bradykinesia in essential tremor?. Eur J Neurol.

[42] Paparella G, Ferrazzano G, Cannavacciuolo A (2018). Differential effects of propranolol on head and upper limb tremor in patients with essential tremor and dystonia. J Neurol.

[43] Paparella G, Angelini L, De Biase A (2021). Clinical and Kinematic Features of Valproate-Induced Tremor and Differences with Essential Tremor. Cerebellum.

[44] De Biase A, Paparella G, Angelini L (2022). Tremor and Movement Slowness Are Two Unrelated Adverse Effects Induced by Valproate Intake. Mov Disord Clin Pract.

[45] Angelini L, Paparella G, De Biase A (2023). Longitudinal study of clinical and neurophysiological features in essential tremor. Eur J Neurol.

[46] Passaretti M, De Biase A, Paparella G (2023). Worsening of Essential Tremor After SARS-CoV-2 Infection. Cerebellum.

[47] Klebe S, Stolze H, Grensing K (2005). Influence of alcohol on gait in patients with essential tremor. Neurology.

[48] Kovalenko E, Talitckii A, Anikina A (2021). Distinguishing Between Parkinson’s Disease and Essential Tremor Through Video Analytics Using Machine Learning: A Pilot Study. IEEE Sens J.

[49] Williams S, Shepherd S, Fang H (2019). Computer vision of smartphone video has potential to detect functional tremor. J Neurol Sci.

[50] van den Noort JC, Kortier HG, van Beek N (2016). Measuring 3D Hand and Finger Kinematics-A Comparison between Inertial Sensing and an Opto-Electronic Marker System. PLoS ONE.

[51] Paparella G, Cannavacciuolo A, Angelini L (2023). May Bradykinesia Features Aid in Distinguishing Parkinson’s Disease, Essential Tremor, And Healthy Elderly Individuals?. J Parkinsons Dis.

[52] Kanko RM, Laende EK, Davis EM (2021). Concurrent assessment of gait kinematics using marker-based and markerless motion capture. J Biomech.

[53] Haubenberger D, Kalowitz D, Nahab FB (2011). Validation of digital spiral analysis as outcome parameter for clinical trials in essential tremor. Mov Disord.

[54] Hess CW, Hsu AW, Yu Q (2014). Increased variability in spiral drawing in patients with functional (psychogenic) tremor. Hum Mov Sci.

[55] Michalec M, Hernandez N, Clark LN, Louis ED (2014). The spiral axis as a clinical tool to distinguish essential tremor from dystonia cases. Parkinsonism Relat Disord.

[56] Ferleger BI, Sonnet KS, Morriss TH (2020). A tablet- and mobile-based application for remote diagnosis and analysis of movement disorder symptoms. Annu Int Conf IEEE Eng Med Biol Soc.

[57] Peters J, Motin MA, Perju-Dumbrava L (2021). Computerised analysis of writing and drawing by essential tremor phenotype. BMJ Neurol Open.

[58] Smits EJ, Tolonen AJ, Cluitmans L (2014). Standardized Handwriting to Assess Bradykinesia, Micrographia and Tremor in Parkinson’s Disease. PLoS ONE.

[59] Louis ED, Gillman A, Boschung S (2012). High Width Variability during Spiral Drawing: Further Evidence of Cerebellar Dysfunction in Essential Tremor. Cerebellum.

[60] Suppa A, Asci F, Saggio G (2021). Voice Analysis with Machine Learning: One Step Closer to an Objective Diagnosis of Essential Tremor. Mov Disord.

[61] Lundy DS, Roy S, Xue JW (2004). Spastic/spasmodic vs. tremulous vocal quality: motor speech profile analysis. J Voice.

[62] Snow G, Guardiani E (2019). Movement Disorders and Voice. Otolaryngol Clin North Am.

[63] de Moraes BT, de Biase NG (2016). Laryngoscopy evaluation protocol for the differentiation of essential and dystonic voice tremor. Braz J Otorhinolaryngol.

[64] Nisticò R, Pirritano D, Salsone M (2012). Blink reflex recovery cycle in patients with dystonic tremor: a cross-sectional study. Neurology.

[65] Arabia G, Lupo A, Manfredini LI (2018). Clinical, electrophysiological, and imaging study in essential tremor-Parkinson’s disease syndrome. Parkinsonism Relat Disord.

[66] Nisticò R, Pirritano D, Novellino F (2012). Blink reflex recovery cycle in patients with essential tremor associated with resting tremor. Neurology.

[67] Gövert F, Becktepe J, Balint B (2020). Temporal discrimination is altered in patients with isolated asymmetric and jerky upper limb tremor. Mov Disord.

[68] Tinazzi M, Fasano A, Di Matteo A (2013). Temporal discrimination in patients with dystonia and tremor and patients with essential tremor. Neurology.

[69] Conte A, Ferrazzano G, Belvisi D (2018). Somatosensory temporal discrimination in Parkinson’s disease, dystonia and essential tremor: Pathophysiological and clinical implications. Clin Neurophysiol.

[70] Münchau A, Schrag A, Chuang C (2001). Arm tremor in cervical dystonia differs from essential tremor and can be classified by onset age and spread of symptoms. Brain.

[71] Britton TC, Thompson PD, Day BL (1993). Modulation of postural wrist tremors by magnetic stimulation of the motor cortex in patients with Parkinson’s disease or essential tremor and in normal subjects mimicking tremor. Ann Neurol.

[72] Lu M-K, Chiou S-M, Ziemann U (2015). Resetting tremor by single and paired transcranial magnetic stimulation in Parkinson’s disease and essential tremor. Clin Neurophysiol.

[73] Pattamon P, Cho HJ, Srivanitchapoom P, Hallett M (2016) Resetting tremor by single pulse transcranial magnetic stimulation of the motor cortex and the cerebellum in essential tremor and dystonic tremor (P4.299). Neurology 86.16 Supplement (2016):P4.299. Web. 03 Oct. 2023. Available online at: https://n.neurology.org/content/86/16_Supplement/P4.299

[74] Benamer TS, Patterson J, Grosset DG (2000). Accurate differentiation of parkinsonism and essential tremor using visual assessment of [123I]-FP-CIT SPECT imaging: the [123I]-FP-CIT study group. Mov Disord.

[75] Menéndez-González M, Tavares F, Zeidan N, Salas-Pacheco JM, Arias-Carrión O (2014). Diagnoses behind patients with hard-to-classify tremor and normal DaT-SPECT: a clinical follow up study. Front Aging Neurosci.

[76] Lorberboym M, Treves TA, Melamed E (2006). [123I]-FP/CIT SPECT imaging for distinguishing drug-induced parkinsonism from Parkinson’s disease. Mov Disord.

[77] Roberts EA, Schilsky ML, American Association for Study of Liver Diseases (AASLD) (2008). Diagnosis and treatment of Wilson disease: an update. Hepatology.

[78] Gajos A, Bogucki A, Schinwelski M (2010). The clinical and neuroimaging studies in Holmes tremor. Acta Neurol Scand.

[79] Ohta E, Takiyama Y (2012). MRI Findings in Neuroferritinopathy. Neurol Res Int.

[80] Lee JH, Yun JY, Gregory A, Hogarth P, Hayflick SJ (2020). Brain MRI Pattern Recognition in Neurodegeneration With Brain Iron Accumulation. Front Neurol.

[81] Ganaraja VH, Holla VV, Stezin A (2022). Clinical, Radiological, and Genetic Profile of Spinocerebellar Ataxia 12: A Hospital-Based Cohort Analysis. Tremor Other Hyperkinet Mov (N Y).

[82] Saleem S, Aslam HM, Anwar M (2013). Fahr’s syndrome: literature review of current evidence. Orphanet J Rare Dis.

[83] Brown SSG, Stanfield AC (2015). Fragile X premutation carriers: A systematic review of neuroimaging findings. J Neurol Sci.

[84] Samuel M, Torun N, Tuite PJ (2004). Progressive ataxia and palatal tremor (PAPT): clinical and MRI assessment with review of palatal tremors. Brain.

[85] Filip P, Vojtíšek L, Baláž M (2020). Differential diagnosis of tremor syndromes using MRI relaxometry. Parkinsonism Relat Disord.

[86] Cherubini A, Nisticó R, Novellino F (2014). Magnetic resonance support vector machine discriminates essential tremor with rest tremor from tremor-dominant Parkinson disease. Mov Disord.

[87] Prodoehl J, Li H, Planetta PJ (2013). Diffusion Tensor Imaging of Parkinson’s Disease, Atypical Parkinsonism and Essential Tremor. Mov Disord.

[88] Bienes GHAA, Zorzenon CDPF, Alves ED (2021). Neuroimaging Assessment of Nigrosome 1 with a Multiecho Gre Magnetic Resonance Sequence in the Differentiation Between Parkinsons Disease from Essential Tremor and Healthy Individuals. Tremor Other Hyperkinet Mov (N Y).

[89] Wang J, Huang Z, Li Y (2019). Neuromelanin-sensitive MRI of the substantia nigra: An imaging biomarker to differentiate essential tremor from tremor-dominant Parkinson’s disease. Parkinsonism Relat Disord.

[90] Doepp F, Plotkin M, Siegel L (2008). Brain parenchyma sonography and 123I-FP-CIT SPECT in Parkinson’s disease and essential tremor. Mov Disord.

[91] Novellino F, Arabia G, Bagnato A (2009). Combined use of DAT-SPECT and cardiac MIBG scintigraphy in mixed tremors. Mov Disord.

[92] Bédard P, Panyakaew P, Cho H-J (2022). Multimodal imaging of essential tremor and dystonic tremor. Neuroimage Clin.

[93] Belenky V, Stanzhevsky A, Klicenko O, Skoromets A (2018). Brain positron emission tomography with 2–18F-2-deoxi-D-glucose of patients with dystonia and essential tremor detects differences between these disorders. Neuroradiol J.

[94] Buijink AWG, van Rootselaar A-F, Helmich RC (2022). Connecting tremors – a circuits perspective. Curr Opin Neurol.

[95] van den Berg KRE, Helmich RC (2021). The Role of the Cerebellum in Tremor - Evidence from Neuroimaging. Tremor Other Hyperkinet Mov (N Y).

[96] Dirkx MF, Zach H, van Nuland A (2019). Cerebral differences between dopamine-resistant and dopamine-responsive Parkinson’s tremor. Brain.

[97] He R, Qin Y, Zhou X (2022). The effect of regional white matter hyperintensities on essential tremor subtypes and severity. Front Aging Neurosci.

[98] Caligiuri ME, Arabia G, Barbagallo G (2017). Structural connectivity differences in essential tremor with and without resting tremor. J Neurol.

[99] Benito-León J, Louis ED, Mato-Abad V (2019). A data mining approach for classification of orthostatic and essential tremor based on MRI-derived brain volume and cortical thickness. Ann Clin Transl Neurol.

[100] de Lima XL, Simonyan K (2020). Neural Representations of the Voice Tremor Spectrum. Mov Disord.

[101] Cerasa A, Nisticò R, Salsone M (2014). Neuroanatomical correlates of dystonic tremor: a cross-sectional study. Parkinsonism Relat Disord.

